# Kiwifruit Alleviates Learning and Memory Deficits Induced by Pb through Antioxidation and Inhibition of Microglia Activation In Vitro and In Vivo

**DOI:** 10.1155/2017/5645324

**Published:** 2017-03-12

**Authors:** Wei-Zhen Xue, Qian-Qian Yang, Yiwen Chen, Rong-Xin Zou, Dong Xing, Yi Xu, Yong-Sheng Liu, Hui-Li Wang

**Affiliations:** ^1^School of Food Science and Engineering, Hefei University of Technology, Hefei, Anhui 230009, China; ^2^Hefei No. 8 High School, Hefei 230038, China

## Abstract

Lead (Pb) exposure, in particular during early postnatal life, increases susceptibility to cognitive dysfunction and neurodegenerative outcomes. The detrimental effect of Pb exposure is basically due to an increasing ROS production which overcomes the antioxidant systems and finally leads to cognitive dysfunction. Kiwifruit is rich in the antioxidants like vitamin C and polyphenols. This study aims to investigate the effects and mechanism of kiwifruit to alleviate learning and memory deficits induced by Pb exposure. Sprague-Dawley (SD) rat pups acquired Pb indirectly through their mothers during lactation period and after postnatal day 21 (PND21) directly acquired Pb by themselves. Five kinds of kiwifruits were collected in this study and the amounts of vitamin C and polyphenols in them were measured and the antioxidation effects were determined. Among them, Qinmei kiwifruit (Qm) showed the strongest antioxidation effects in vitro. In vivo, Qm significantly repaired Pb-induced learning and memory deficits and dendritic spine loss. In addition, Pb compromised the enzymatic activity and transcriptional levels of SOD and GSH-Px and decreased the microglial activation, which, to some extent, could be reversed by Qm kiwifruit administration. The results suggest that kiwifruit could alleviate Pb-induced cognitive deficits possibly through antioxidative stress and microglia inactivation. Consequently, kiwifruit could be potentially regarded as the functional food favorable in the prevention and treatment of Pb intoxication.

## 1. Introduction

The* Actinidia Chinensis*, commonly known as kiwifruit, has been praised as “the king of fruits” based on its remarkable abundance of vitamin C, polyphenols, and other health-beneficial metabolites. In addition, 94% of vitamin C in kiwifruit can be absorbed by human beings. Extensive studies on antioxidation in vivo and in vitro have been reported in kiwifruit [1, 2].

Heavy metals, such as Pb, are naturally occurring because they are released by natural events and human activities. Even though in developed countries human exposure to toxic metals is decreasing, in developing countries it is increasing. Pb can cause cognitive impairment and is generally considered to be a high risk factor for attention-deficit hyperactivity disorder (ADHD) [3] and Alzheimer's disease (AD) [4]. It was well-documented that low level Pb exposure could still impair the learning and memory ability. In accordance with its toxic property, the United States CDC Advisory Committee states that “no level of Pb appears to be safe” [5].

The deleterious impacts of Pb on intelligence have evoked some experimental studies to clarify its neurotoxic mechanism. Oxidative stress has been demonstrated as one of the crucial mechanisms of cognitive deficits of Pb [6, 7]. Oxidative stress is an unbalanced status between the generation of reactive oxygen species (ROS) and antioxidant defense system. Pb-induced oxidative stress results in the enhanced production of ROS, which may induce tissue damage and cognitive impairments in animal models [8–11]. Mechanistically, microglia plays an important role in synaptic plasticity, possibly through release of cytokines and growth factors [12]. Of note, oxidative stress is considered as a causative agent leading to microglia activation [13, 14]. Recent study showed that Pb neurotoxicity may also be mediated by microglia activation, which induces high-level expression of many cytokines, such as TNF-*α* and IL-1*β*, thus giving rise to learning and memory deficits [15].

In the present study, we aimed to investigate the effect of kiwifruit administration on Pb-induced learning and memory deficits in rats and explored the possible molecular mechanism, mainly focusing on antioxidation and microglial activation.

## 2. Materials and Methods

### 2.1. Pretreatment of Kiwifruit

Five cultivars of kiwifruit (Hayward, Xuxiang, Qinmei, Jinkui, and Wancui) were supplied by Professor Liu et al. [6]. All kiwifruits were prepared using fruit squeezer following peeling. The kiwifruit juice was mixed with 60% ethanol (v : v = 1 : 2) and extracted at 40°C by sonication for 20 min. After centrifugation for 20 min at 4000 rpm, supernatant was collected as the fresh kiwifruit juice. In order to ensure that the components of kiwifruit were the same in further experiments, the fresh kiwifruit juice was concentrated by vacuum rotatory evaporator at 45°C and lyophilized to yield the crude kiwifruit powder and stored at −80°C for the further experiments.

### 2.2. Bioactive Components Contents

The total phenolic content of five fresh kiwifruit juice was determined by Folin-Ciocalteu method. Briefly, 0.5 mL dissolved juice was mixed with 0.4 mL of 50% Folin-Ciocalteu reagent, 0.8 mL of 20%  Na_2_CO_3_. After incubation at 37°C for 2 h, the absorbance of the reaction mixture was measured at 760 nm using a Microplate Reader (Thermo, Multiskan GO). Gallic acid was used as a standard, and the total polyphenols content of kiwifruit was examined by milligram gallic acid equivalents per liter juice (mg GAE/L juice).

And the main component, vitamin C, in fresh kiwifruit juice and kiwifruit powder was resolved on a high performance liquid chromatography (HPLC) equipment of Waters e2695 with a 2998 photodiode array detector. Samples (20 *μ*L) were separated on a Symmetry C18 column (250 mm × 4.6 mm, 5 *μ*m). The mobile phase consisted of methyl alcohol, acetonitrile, and 0.02% phosphoric acid (5 : 10 : 85; v : v : v). The flow rate was 1.0 mL/min, and the column temperature was 30°C. The UV-Vis chromatograms were recorded at 240 nm.

### 2.3. Total Antioxidant Activity In Vitro

Total antioxidant activity of five cultivars of kiwifruit powder was first assessed on the basis of the radical scavenging effect of the 1,1-diphenyl-2-picrylhydrazyl- (DPPH-) free radical activity [7]. Different concentration of different species of kiwifruit powder or vitamin C (the positive control) was prepared in methanol. 60 *μ*L of different concentration of samples was taken separately and mixed with 120 *μ*L of 62.5 *μ*M DPPH solution (A). After incubation in dark for 30 min, the absorbance was taken on Microplate Reader at 517 nm. The radical scavenging activity (Inhibition%) was calculated using the following formula. The FRAP assay was also employed to evaluate the ability of antioxidation and applied with minor modification as described by Oikeh et al. [8]. The method is based on the reduction of a ferric 2,4,6-tripyridyl-s-triazine complex (Fe^3+^-TPTZ) to the ferrous form (Fe^2+^-TPIZ). To conduct the assay, 180 *μ*L TPIZ working solution mixed with 0.3 M acetate buffer, 10 mM TPTZ in 40 mM HCL, and 20 mM ferric chloride (10 : 1 : 1 v/v/v) were combined with 10 *μ*L of different concentration of powder or vitamin C. After incubation at 37°C for 30 min, the absorbance of the reaction mixtures was measured at 593 nm by a Microplate Reader. To determine the antioxidant capacity of different samples, the absorbance values were compared with those obtained from the standard curves of FeSO_4_. The antioxidant capacity values were expressed as millimole of FeSO_4_ equivalent in 10 mg kiwifruit powder.

### 2.4. Cell Line and Cell Culture

The PC12, a rat pheochromocytoma cell line, was originally procured from University of Science and Technology of China (Hefei, China). The cells were cultured in 89% DMEM (HyClone, USA), supplemented with 10% heat-inactivated FBS (WISENT, CAN) and 1% 100x Penicillin-Streptomycin Solution (Sigma, USA). Cells were maintained in a humidified atmosphere of 95% air and 5% CO_2_ at 37°C in an incubator (Thermo HERAcell 150i).

### 2.5. Oxidative Stress Evaluation in PC12 Cells

The cell viability was estimated by 3-(4,5-dimethylthiazol-2-yl)-2, 5-diphenyltetrazolium bromide (MTT) assay. PC12 cells were seeded in 96-well plate at a concentration of 2 × 10^4^ cells/well. After 12 h, the cells were, respectively, treated with different concentrations of Pb (2.5–50 *μ*M), vitamin C (100 *μ*M), and different kiwifruit powder (12 mM, the content of vitamin C is equal to the positive control), and incubated in the incubator for 24 h. Thereafter, 50 *μ*L of MTT-PBS solution (5 mg/mL) was added and incubated for 4 h. The supernatants were aspirated and the formazan crystals in each well were dissolved in 50 *μ*L of DMSO. Absorbance was measured by Microplate Reader at a wavelength of 570 nm. Relative cell viability was determined by the amount of MTT converted to the formazan. The optical density of the formazan formed in the control cells was calculated as 100% viability.

Intracellular formation of ROS in PC12 cells which was assessed using oxidation sensitive dye 2′,7′-dichlorodihydro fluorescein diacetate (DCFH-DA; Sigma-Aldrich, USA) as the fluorescence agent [9]. Briefly, cells were seeded in 24-well plate which was plated with poly-L-lysine-precoated tissue culture slides. Cells were then, respectively, treated with vitamin C (100 *μ*M), kiwifruit powder (12 mM), Pb (10 *μ*M), Pb + vitamin C (100 *μ*M), and Pb + kiwifruit powder (12 mM) and incubated for 24 h. Then cells were washed twice with 1 × PBS and incubated for 30 min with 20 *μ*M DCFH-DA at 37°C in the dark. The supernatants were discarded and slides were washed three times with serum-free medium and observed using an upright fluorescence microscope (Nikon Eclipse 80i, Japan) using a 10x objective. Quantification of fluorescence was done using the Image J analysis software.

### 2.6. Experimental Animals

Sprague-Dawley (SD) rats were obtained from the Laboratory Animal Center, Anhui Medical University, China. Rats were fed with laboratory chow and distilled water and individually housed in an ambient temperature (20 ± 2°C) and relative humidity (50 ± 10%) controlled environment on a 12 h-12 h light-dark cycle with laboratory chow and distilled water. All experimental operations comply with the National Institute of Health Guide for the Care and Use of Laboratory Animals and were approved by institutional animal care and use committee of Hefei University of Technology, China. The rat pups were randomly divided into six groups as follows: (1) control; (2) control + vitamin C 100 mg kg^−1^; (3) control + kiwifruit powder 12 g kg^−1^; (4) Pb exposed; (5) Pb exposed + vitamin C 100 mg kg^−1^; (6) Pb exposed + kiwifruit powder 12 g kg^−1^. (*n* = 10 rats in each group).

**Figure 1:**
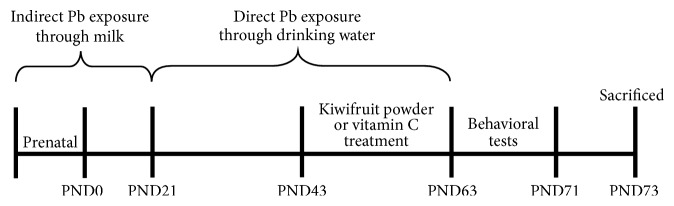
The schedule of in vivo experiments.

The protocol for exposure to Pb has been reported previously [14]. The pups acquired Pb indirectly through their mothers and after postnatal day 21 (PND21) directly acquired Pb by themselves. From 7 weeks to 9 weeks old, the pups were treated with vitamin C or kiwifruit powder which was received daily intragastric gavage. The vitamin C and kiwifruit powder were dissolved in physiological saline. At the same period, the control group and the Pb-exposed group were administrated daily with physiological saline alone. After the last treatment period, the rats were given one day of rest and subjected to behavioral tests. Then all the rats were sacrificed by CO_2_ and brain tissues were collected for subsequent experiments (Figure 1).

### 2.7. Behavioral Tests

Rats in the experiment underwent two behavioral tests including the Y-maze and Morris water maze (MWM). In a spontaneous alternation of tests, rats were allowed to move freely through the Y-maze for 5 min. Alternation was defined as successive entries into the three arms of an overlapping triplet set. The percentage of alternation was calculated as the total number of alternations × 100/(total number of arm entries-2) [16]. The MWM experiments were performed in a circular pool with a diameter of 160 cm and depth of 70 cm. It filled to a depth of 40 cm with opaque water by addition of caramel coloring, keeping the temperature about 23 ± 1°C. Each rat was trained for four trials daily for 5 days to find the hidden platform. When it found the platform, it had 30 s to stay on it. If it failed to reach the platform within 60 seconds, it was guided and allowed to remain there for the same period of time. The platform was removed on the sixth day; then each rat was afforded 60 s probe trial to measure its faculty of memory retention. Performance was video-recorded and analyzed by image analyzing software (ANY-maze; Stoelting Co., Ltd, USA). The platform crossings and time spent on the target quadrant were recorded.

### 2.8. Measurement of Pb and Vitamin C Tissue Incorporation

The concentration of Pb in hippocampus of the rats was determined using a graphite furnace atomic absorption spectrometry [11]. Appropriate hippocampus (0.05–0.3 g) was mixed with 2 mL of 30% hydrogen peroxide (AR, Sinopharm Chemical Reagent Co., Ltd.) and 4 mL of nitric acid (GR, Sinopharm Chemical Reagent Co., Ltd.) standing overnight. The mixture was digested at 200°C for 30 min in a microwave nitrate pyrolysis furnace (EMR Marsxpress Certificate, VB 20), evaporated, and diluted with double distilled water to 5 mL. The concentration of Pb was determined by a PerkinElmer Analyst 800 spectrometer.

Measurement of vitamin C in hippocampus was performed by HPLC-ECD analysis. 1 g of brains obtained from kiwifruit powder treatment group (*n* = 8 rats) was grinded in mortar with 1 mL of cold phosphate-buffered saline (PBS, pH 7.4). The hippocampus was transferred into a tube to be homogenized by sonication (50 W × 15 s) on ice. The organic impurity was removed by 1.5 mL HPLC-grade hexane (Sinopharm Chemical Reagent Co., Ltd.), and then the water layer was filtered. Vitamin C was separated under isocratic condition using methyl alcohol, acetonitrile, and 0.02% phosphoric acid (5 : 10 : 85; v : v : v) (Sinopharm Chemical Reagent Co., Ltd.) and a column (Symmetry C18, 5 *μ*m, 4.6 × 250 mm). The absorbance of the vitamin C was measured at 240 nm. Compound identification and analysis calibration were based on use of vitamin C (Sinopharm Chemical Reagent Co., Ltd.) as external standards.

### 2.9. Golgi-Cox Staining and Spine Density Assay

To analyze the changes in the morphology of the dendritic spines in hippocampus, the Golgi-Cox staining was applied with minor modification as described by Hu et al. [12]. Briefly, brains stored at 37°C in dark place for two days in Golgi-Cox solution were sectioned at 200 *μ*m in 6% sucrose with a vibratome (VT1000S, Leica, GER). All sections were collected on 2% gelatin-coated slides. Then slices were stained with ammonia for 60 min, washed with water for three times, followed by Kodak Film Fix for 30 min, and then washed with water, dehydrated, cleared, and mounted using a resinous medium. The pyramidal neurons in hippocampal region were imaged with a Nikon microscope (Nikon Eclipse 80i, Japan) using a 40x objective. The spines counted in the present study were on 2-3 stretches of the secondary dendrite about 10 *μ*m in length. About 10–15 neurons from one animal were selected to quantify the spine density. Generally, brains were longitudinally cut into two halves and one hemisphere was processed for morphological staining and the other hemisphere was used to examine special proteins and genes expression.

### 2.10. Oxidative Stress Evaluation in Rats

The activity of SOD and GSH-Px was measured to assess the level of oxidative stress in hippocampus. SOD and GSH-Px detection kits (Jiancheng, CHN) were used, and procedures were carried out according to the instructions of the manufacturer.

### 2.11. Florescent Quantitative Real-Time PCR

The florescent quantitative real-time PCR was performed to analyze the mRNA levels of SOD2, GSH-Px, TNF-*α*, and IL-1*β* in rats. First total RNAs were extracted from hippocampus samples using the AxyPrep Multisource Total RNA Miniprep Kit (Axygen, USA). Subsequently, the reverse transcription reaction was completed according to the manufacturer's instruction (TransGen, CHN), resulting in the first strand of total cDNA. Quantitative PCR was performed on Light Cycler 96 (Roche, CH) using primers pairs listed in Table 1. The 20 *μ*L reaction pool of qPCR was composed of 10 *μ*L of SYBR premix Ex-Taq; 0.6 *μ*L of forward and reverse primer each; 1 *μ*L of cDNA template (10 times dilution) and 7.8 *μ*L of deionized water. The reaction protocol was set as one cycle of 95°C 5 s, 60°C 30 s, followed by the dissociation stage of 95°C 15 s, 60°C 30 s, and 95°C 15 s. The results were normalized against *β*-actin as an internal control. Each target gene was performed four times.

### 2.12. Western Blot Analysis

Hippocampal tissues were homogenized and dissolved in the ice lysis-buffer containing a cocktail of protein phosphatase and protease inhibitors (21 *μ*g/mL aprotinin, 0.5 *μ*g/mL leupetin, 4.9 mM MgCl_2_, 1 mM sodium-Meta-vanandante, 1% Triton X-100, and 1 mM PMSF) to avoid dephosphorylation and degradation of proteins. The samples were centrifuged at 14000 rpm at 4°C for 7 min. The total protein of supernatant was quantified using the bicinchoninic acid (BCA) protein assay (Beyotime Biotechnology, CHN). 25 *μ*g of proteins was loaded on 12% SDS-PAGE gel for electrophoresis, and separated proteins were transferred to PVDF membrane (Millipore, USA), blocked with 5% nonfat dry milk, followed by incubation with primary antibodies overnight at 4°C. Then membranes were washed for three times, incubated with secondary antibody, and developed using the enhanced chemiluminescence immuno-blotting detection system. The antibody Iba1 was purchased from Abcam (Cambridge, UK). The band intensity was normalized to *β*-actin (Abcam, UK) when analyzing.

### 2.13. Statistical Analysis

All data were expressed as mean ± SEM. One-way ANOVA and *T*-test were applied to statistical analyses of most of the experiments. Two-way ANOVA was only used to the data of training in Morris water maze. Difference between experiment groups was tested by Fisher's protected least significant difference (PLSD) with 95% confidence. A value of *p* < 0.05 was considered to be statistically significant.

## 3. Results

### 3.1. Vitamin C and Polyphenols Contents in Kiwifruit

**Figure 2:**
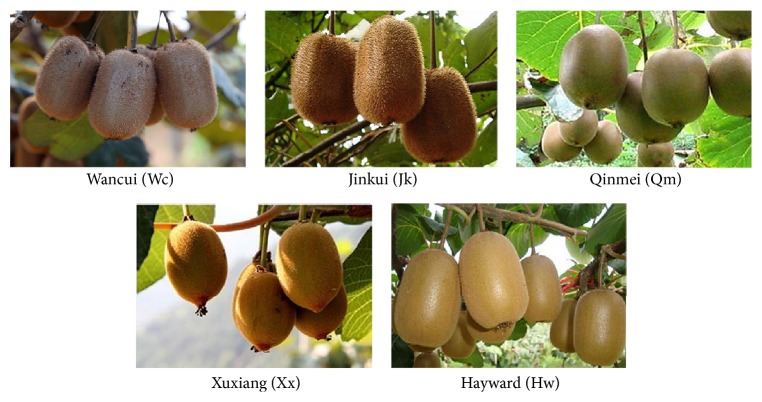
The five cultivars of kiwifruit in the study.

**Figure 3:**
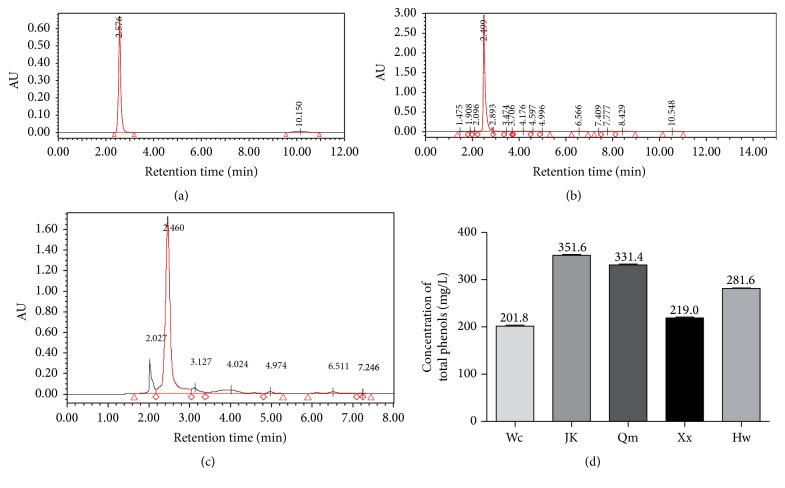
Measurements of the bioactive components in five cultivars of kiwifruit. The chromatogram of standard vitamin C (a), fresh kiwifruit juice (b), and kiwifruit powders after pretreatment (c). (d) Histograms were quantitatively analyzed for polyphenol contents by Folin-Ciocalteu in five kiwifruit powders after pretreatment.

There were five kinds of commonly consumed fresh kiwifruit, named as Wancui, Jinkui, Qinmei, Xuxiang, and Hayward (Figure 2), used in this study. As phenolic compounds act as the important reducing agents and antioxidants in kiwifruit, the total polyphenols in five kiwifruit juice were tested from regression equations of calibration curves and were expressed in gallic acid equivalent [13]. As shown in Figure 3(d) Jinkui kiwi (351.6 ± 1.36 mg GAE/g powder) and Qinmei kiwi (3314 ± 3.28 mg GAE/g powder) contained more polyphenols than the others.

Vitamin C was considered as the predominant antioxidant among various components of kiwifruit. Thus, the amount of vitamin C was subsequently determined by HPLC (Figure 3). As Table 2 showed, each kiwifruit has different amount of vitamin C. In the fresh juice, Wancui kiwi (0.29 ± 0.01 g/L) and Jinkui kiwi (0.30 ± 0.02 g/L) contained higher amount of vitamin C than others. However, in the powder, Qinmei kiwi is rich in vitamin C the most (0.84 ± 0.18%). Besides, the loss of its vitamin C through pretreatment was 18.73 ± 0.60%, suggesting that Qinmei kiwi remained rather stable during the process.

### 3.2. Total Antioxidant Activity of Kiwifruit In Vitro

**Figure 4:**
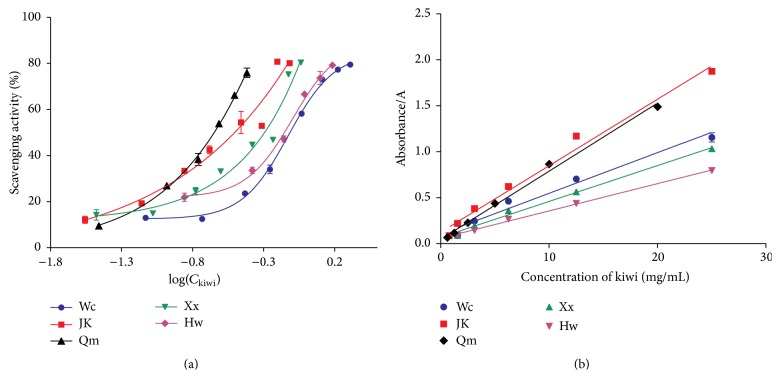
The antioxidant properties of five kiwifruits were evaluated in vitro. Graph shows the DPPH radical scavenging activity (a) and FRAP assay (b) of five kiwifruit powders after pretreatment. The experiments were repeated at least three times.

To examine the antioxidant activity of different kiwifruits, we tested antioxidant effects in vitro using DPPH and FRAP methods. DPPH is a common method used to assess the free radical scavenging activity of many antioxidant substances [14].  Figure 4(a) indicated that all kiwifruit possessed antioxidant activities and different cultivars differed in their performances in DPPH radical scavenging. Qinmei kiwi showed the highest DPPH radical scavenging activity, with its EC_50_ value reaching 0.40 ± 0.01 mg/mL (Table 3). The EC_50_ value of positive control, namely vitamin C was 5.61 ± 0.25 *μ*g/mL. 0.4 mg Qinmei powder contained about 3.36 *μ*g Vc, less than the EC_50_ value of Vc (5.61 ± 0.25 *μ*g/mL). So our result showed Qinmei kiwi has more powerful DPPH radical scavenging activities than the positive control. The FRAP assay is also performed as routine analysis to assess the antioxidant ability due to its simplicity, rapidity, and sensitivity. The ability to decrease the TPTZ-Fe (III) to a TPIZ-Fe (II) of different kiwifruit juice was shown in Figure 4(b). These results suggested kiwifruit of Qinmei and Jinkui has a similar FRAP antioxidant ability compared with the other cultivars of kiwifruits (Table 4). The antioxidant of different kiwifruit was consistent with amounts of vitamin C and total polyphenols.

Based on the results above, as Qm showed the best antioxidant effect, we chose the kiwifruit of Qinmei as the optional candidate for further study.

### 3.3. Inhibitory Effects of Kiwifruit on Pb-Induced Oxidative Stress in PC12 Cells

**Figure 5:**
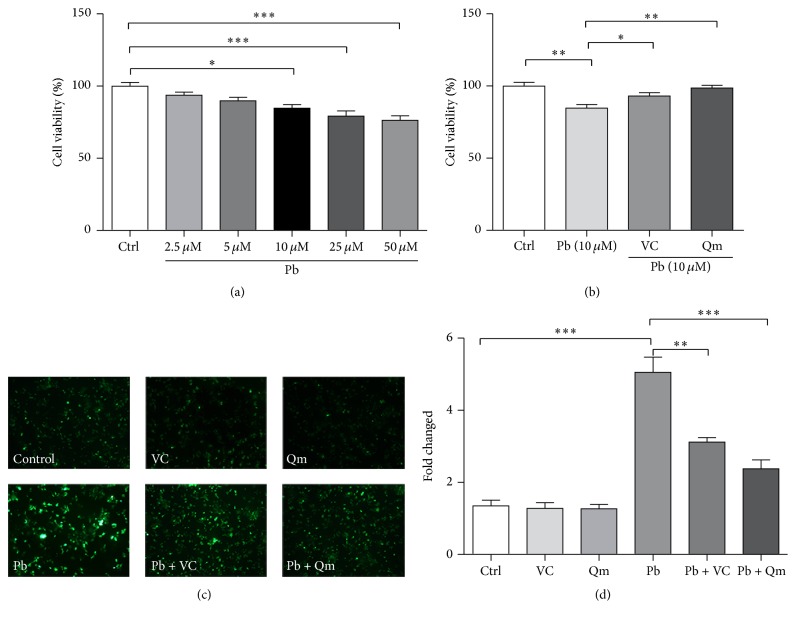
Effects of kiwifruit treatment on Pb-induced oxidative stress in PC12 cells. (a) PC12 cells viability was assessed by MTT test after being treated with various concentrations of Pb (2.5, 5, 10, 25, and 50 *μ*M) for 24 h. The data were analyzed by using one-way-ANOVA, followed by LSD test. (b) PC12 cells were treated with Pb, kiwifruit (Qm), and the positive control (vitamin C) for 24 h, and cell viability was assessed; We compared group of Pb, Vc, and Qm with the control group using *T*-test for two independent samples, respectively. (c) ROS was detected with fluorescence microscope by using fluorescent probe DCFH-DA. (d) Histograms were quantitatively analyzed for changes in fluorescence intensities using the Image J software. The results are expressed as percent control. A representative experiment that was repeated at least three times. We compared group of Ctrl with Pb, Pb + Vc, and Pb + Qm using one-way ANOVA, followed by LSD test. (^*∗*^*p* < 0.05; ^*∗∗*^*p* < 0.01; ^*∗∗∗*^*p* < 0.001.)

Given kiwifruit has strong antioxidant effects in vitro, we wonder if kiwifruit could alleviate oxidative stress induced by Pb exposure. First, we examined the effect of Qinmei kiwi on cell viability in PC12 cells exposed to Pb. As shown in Figure 5(a), compared to the control group, Pb concentration at either 2.5 *μ*M or 5 *μ*M was detected to be noncytotoxic, but the cell viability was significantly reduced by Pb concentration ranging from 10 to 50 *μ*M. Based on principles of low level Pb exposure, 10 *μ*M Pb was applied in further experiments in PC12 cells. And from Figure 5(b), we found that the kiwifruit powder supplement significantly improved the impaired cell viability following Pb exposure.

Excessive ROS was indicated as a signature of oxidative stress. Using DCFH-DA as the fluorescence indicator, it was shown that Pb (10 *μ*M) exposure induced a higher level of ROS production compared with control cells (*p* < 0.001, Figures 5(c) and 5(d)). However, when supplemented with kiwifruit powder (*p* < 0.001) or vitamin C (*p* < 0.05), the cellular ROS levels in cells returned to normal level. The data suggested that Pb-induced oxidative stress could be alleviated by kiwifruit, thereby offering better protection against the Pb-induced neurotoxicity.

### 3.4. Kiwifruit Alleviates Working Memory Deficits Induced by Pb Exposure in SD Rats

Compelling evidence suggested Pb-induced oxidative stress acts as one of the important mechanisms of cognitive deficits [15]. To further identify the effect of kiwifruit on Pb-induced learning and memory deficits, Y-maze and Morris water maze were employed to evaluate the ability of spatial learning and memory in rats.

**Figure 6:**
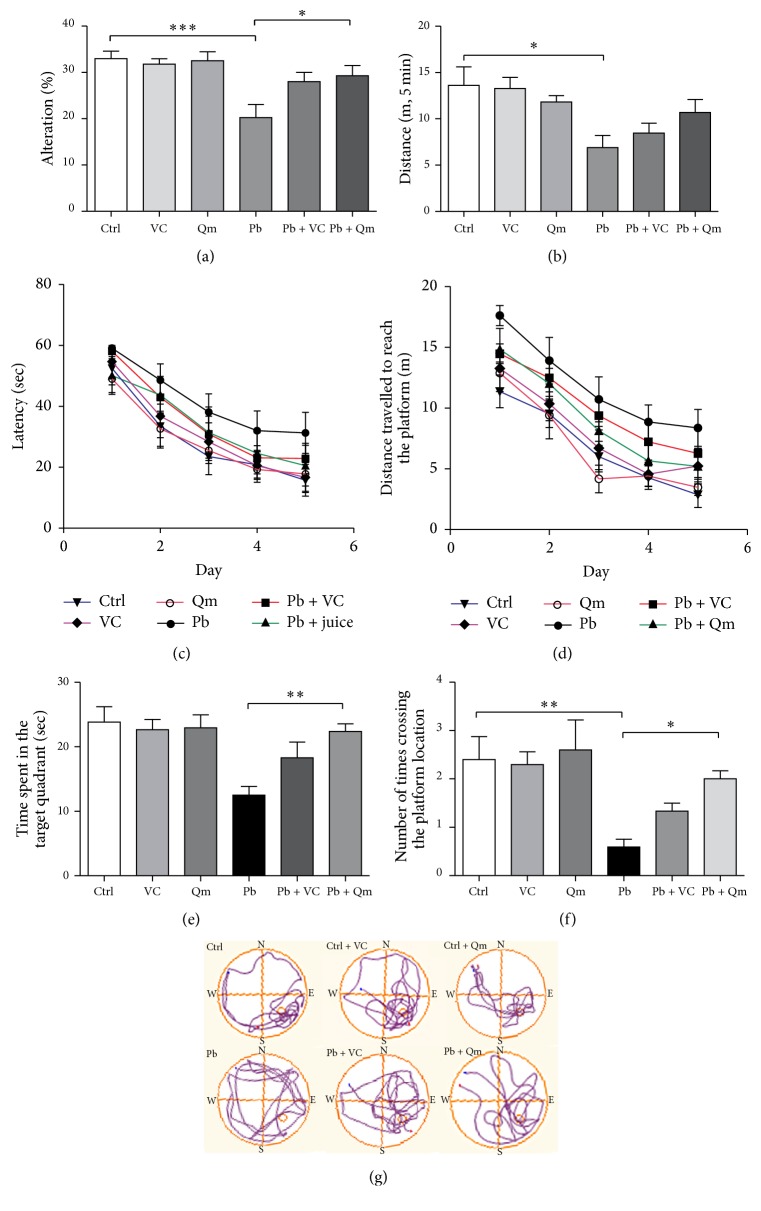
Effects of kiwifruit treatment on Pb-exposed SD rats behavioral performances. Percentage of alternation (a) and distance in 5 min (b) in Y-maze test. For analysis of the Y-maze test's data we used one-way ANOVA, followed by LSD test. Latency (c), distance travelled to reach the platform (d), time percentage in target quadrant (e), and platform crossings (f) by SD rats during MWM training tests, respectively. We compared group of Ctrl with Pb, Pb + Vc, and Pb + Qm using two-way ANOVA with TUKEY test. (g) Representative swimming paths in the probe test of the MWM experiment. The directions “north,” “south,” “east,” and “west” are indicated as “N,” “S,” “E,” and “W,” respectively. The “southeast” quadrant was the target quadrant. There were 10 rats (5 male rats and 5 female rats) in each group. (^*∗*^*p* < 0.05; ^*∗∗*^*p* < 0.01; ^*∗∗∗*^*p* < 0.001.)

In Y-maze test, the rats in kiwifruit powder supplement group performed better than the saline-treated group after Pb exposure (Figures 6(a) and 6(b)). In spontaneous alternation tests, the rats treated with kiwifruit powder showed a higher spontaneous alternation percentage (Figure 6(a)). No significant difference was observed in distance travelled between kiwifruit and saline-treated group following Pb exposure (Figure 5(b)). In MWM tests, the main effect of Pb treatment or training day significantly affected the average latency (*F*(1,85) = 11.70, *p* < 0.01; *F*(4,85) = 19.67, *p* < 0.001) (Figure 5(c)) and distance travelled to the hidden platform (*F*(1,85) = 49.79, *p* < 0.001; *F*(4,85) = 20.06, *p* < 0.001) (Figure 6(d)) and interestingly, the damage induced by Pb exposure could be partly rescued by kiwifruit treatment. Two-way ANOVA also showed the kiwifruit treatment or training day remarkably decreases the latency (*F*(1,90) = 3.69, *p* < 0.05; *F*(4,90) = 18.02, *p* < 0.001) (Figure 6(c)) and distance travelled to the hidden platform (*F*(4,90) = 6.84, *p* < 0.01; *F*(1, 90) = 16.80, *p* < 0.001) (Figure 6(d)). Probe tests showed that Pb exposure decreased the time spent in target quadrant (Figure 6(e), *p* < 0.01) and the times crossing the platform (Figure 6(f), *p* < 0.01). Kiwifruit treatment significantly increased the time spent in the target quadrant and the times crossing the platform compared with Pb-exposed group. Collectively, kiwifruit exerted the neuroprotection effect against Pb-induced spatial memory deficits.

### 3.5. Kiwifruit Increased Dendritic Spine Density in Pb-Exposed Rats

**Figure 7:**
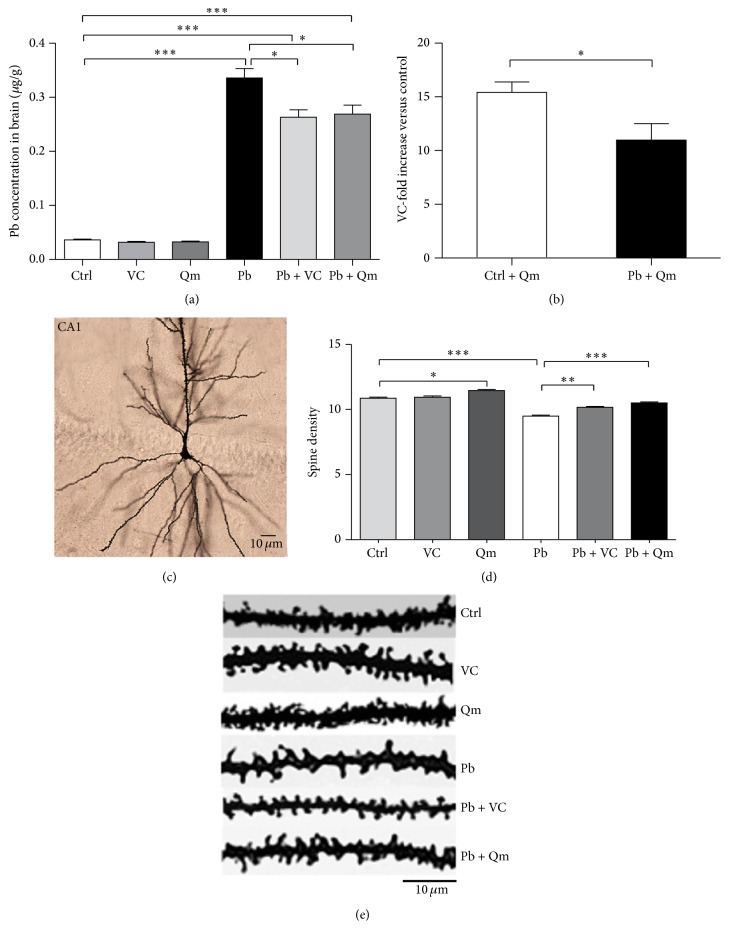
Effects of kiwifruit treatment on dendritic spine density in Pb-exposed rats. (a) Concentration of Pb in brain of rats in different groups. (b) Bioavailability of vitamin C (the main bioactive components of kiwifruit) in brain after systemic treatment and expressed as fold increase compared to untreated rats nonexposed to Pb (control). (c) Golgi-Cox staining represents the dendritic arborization of CA1. The changes of dendritic spine density (the number of dendritic spines contained within 10 *μ*m) (d) and dendritic shaft with spines (e) in CA1 pyramidal neuron. *N* = 6 rats and *n* = 30*～*35 neurons per group. For analysis of these data we used one-way ANOVA, followed by LSD test. (^*∗*^*p* < 0.05; ^*∗∗*^*p* < 0.01; ^*∗∗∗*^*p* < 0.001.)

Figure 7 showed that there was a significant difference in Pb concentration between control and Pb-exposed group (Figure 7(a)). And rats treated with kiwifruit powder (Ctrl + Qm group) exhibited about a 15-fold increase in brain level of kiwifruit powder compared to untreated rats (control group) (Figure 7(b)). That indicated intragastric kiwifruit treatment is able to induce a remarkable brain bioavailability. As the morphological and structural basis for synaptic plasticity, learning, and memory, the dendritic spine densities in CA1 area were markedly reduced in Pb-exposed rats (−12.6%, Figure 7), which was consistent with our previous study [17]. Of note a significant increase of spines, superior to those of vitamin C treated group, was found in presence of kiwifruit powder supplementation following Pb exposure respect to the control group (10.7%, Figure 7(d)). Interestingly, control rats administrated with kiwifruit powder showed a higher density of spines, and there was no striking increment through the sole addition of vitamin C. The result indicated that kiwifruit was able to protect against Pb-induced dendritic spines loss.

### 3.6. Kiwifruit Increased SOD, GSH-Px Activity, and Their mRNA Expression in Pb-Exposed Rats

**Figure 8:**
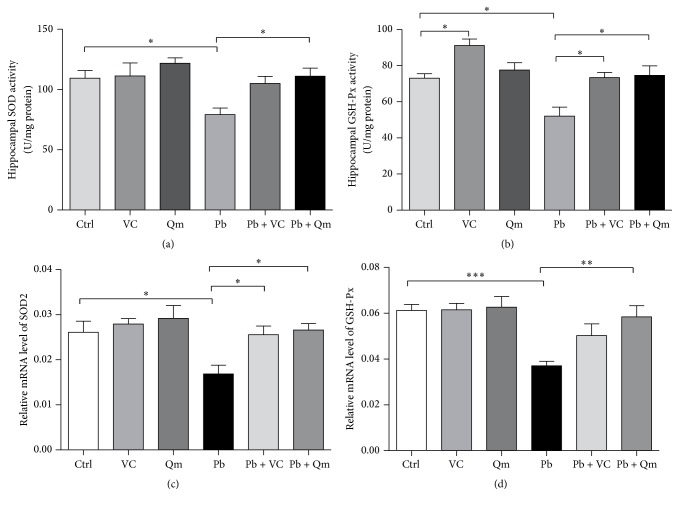
Effects of kiwifruit treatment on selected indicators of oxidative stress in Pb-exposed rats. Effects of kiwifruit on SOD activity (a) and GSH-Px activity (b) in the hippocampus. Effects of kiwifruit on SOD2 (c) and GSH-Px (d) mRNA expression in the hippocampus. *N* = 6 rats per group. For comparing each group we used one-way ANOVA with LSD test. (^*∗*^*p* < 0.05; ^*∗∗*^*p* < 0.01; ^*∗∗∗*^*p* < 0.001.)

Figures 8(a) and 8(b) summarized the selected parameters indicating the extent of oxidative stress in hippocampus. The activity of SOD and GSH-Px in the Pb-exposed group was significantly suppressed compared with the control group, and administration of kiwifruit powder contributed to a significant recovery in SOD and GSH-Px activity in hippocampus. But there was no evident changes of these oxidative stress parameters between control + kiwifruit powder and control groups in hippocampus.

Subsequently, the mRNA level of these relevant proteins in hippocampus by supplementation of kiwifruit powder was also detected by quantitative real-time PCR. As shown in Figures 8(c) and 8(d), consistent with the activity of SOD and GSH-Px, Pb significantly decreased the mRNA level of SOD2 (a manganese-dependent superoxide dismutase) and GSH-Px compared to control group. However, this reduction was reversed by administration of kiwifruit. The current findings suggested a reversed tendency of these oxidative parameters in hippocampus by administration of kiwifruit powder in Pb-exposed group.

### 3.7. Kiwifruit Inhibited Microglia Activation in Pb-Exposed Rats

**Figure 9:**
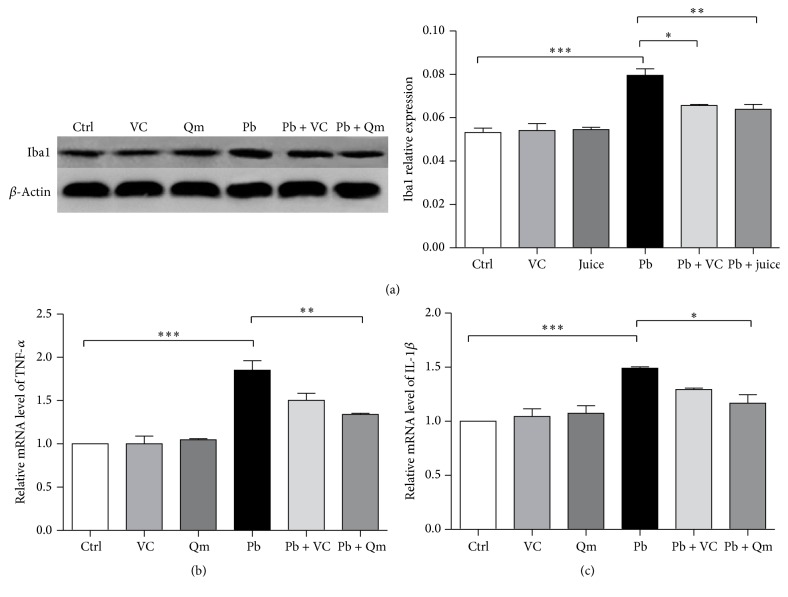
Effects of kiwifruit treatment on neuroinflammatory markers. (a) The representative immunoblot and corresponding densitometric analysis showing Iba1 protein expression in hippocampus. Histograms show the TNF-*α* (b) and IL-1*β* (c) mRNA expression in hippocampus. *β*-Actin was used as a loading control. Iba1 and *β*-actin were run on the same blot. *N* = 4 rats per group. One-way ANOVA was used to analyze 6 groups, followed by LSD test. (^*∗*^*p* < 0.05; ^*∗∗*^*p* < 0.01; ^*∗∗∗*^*p* < 0.001.)

In response to oxidative stress, microglia becomes activated and released cytotoxic factors, like proinflammatory cytokines, and induced serious neurotoxic effects, such as cognition deficit [18–20]. Activated microglia can be quantified using Iba1 marker, which is upregulated during activation [21]. Western blotting assay was applied to determine Iba1 expression level in hippocampus. The results in Figure 9(a) revealed that, in Pb-exposed rats, the expression level of Iba1 was significantly increased, a phenomenon largely reversed by the addition of vitamin C and Qinmei kiwi. Moreover, kiwifruit powder showed better effect than vitamin C on the modulation of Iba1's expression. These results suggested that Pb stimulation caused microglia activation and kiwifruit played an important role in reducing microglia activation and providing central nervous system protection.

### 3.8. Kiwifruit Reduced Inflammatory Factors' Release in Pb-Exposed Rats

Proinflammatory factors have been established as downstream mediators of activated microglia. To further examine the expression of proinflammatory factors, we assessed the transcription levels of TNF-*α* and IL-1*β* in hippocampus by fluorescence quantitative real-time PCR. As shown in Figures 9(b) and 9(c), the expressions of TNF-*α* and IL-1*β* mRNA were greatly increased with the treatment of Pb. Interestingly, kiwifruit powder treatment led to a significant reduction of these inflammatory factors (Figures 9(b) and 9(c)), thus indicating that neuronal inflammatory was reduced by kiwifruit supplementation following Pb exposure.

## 4. Discussion

Pb is a ubiquitous threat to human health. According to the World Health Organization, Pb poisoning accounts for about 0.6% of the global burden of disease [22]. Recent studies have shown that the pathogenesis of Pb toxicity has been associated with oxidative stress [23]. In this study, we showed that kiwifruit alleviated learning and memory deficits elicited by Pb and the effects were primarily attributable to the fine-tuning of antioxidative mechanisms.

Oxidative stress is a process associated with the accumulation of DNA, RNA, and protein damage and a progressive decline in organ function and ultimately results in learning and memory deficits in CNS [15, 24]. Oxidative stress arises when there is an excessive generation of ROS such as oxygen ions or peroxides or reduction in the free radical scavenging machinery such as loss of antioxidant enzymes or other antioxidants [25]. ROS are by-products of organism metabolism and play an important role in cell signaling and homeostasis. However, excessive ROS contributed to the pathogenesis of neurodegenerative disease such as Parkinson's disease and Alzheimer's disease [26, 27]. Thus different antioxidants have been found to possess preventive and therapeutic effects on these diseases [15, 28, 29]. These studies showed the neuroprotective effect of a potent inhibitor of free radical and lipid peroxidation in synaptosome, antioxidant, against oxidative stress in both cell and animal model. Therefore, extensive efforts have been made to recognize both natural and artificial antioxidants and neuroprotective agents in recent years. In this respect, kiwifruit is a promising candidate due to its nature as a nutritive and edible biological substance. However, it was poorly documented that this fruit could efficiently address the serious intellectual issues via the remodeled antioxidative system. This study is also the first to report the inhibitory effects of kiwifruit on microglia activation by Pb exposure.

The kiwifruit is a nutritionally and economically important fruit with remarkably high vitamin C and polyphenols content [6] and thus possessing excellent antioxidative properties [30]. There are different cultivars of kiwifruit in China. In the study, antioxidant effects in vitro were tested by four indicators, the content of vitamin C by HPLC, measurement of total polyphenols by Folin-Ciocalteu, and scavenging DPPH and FRAP. All these indicators suggested that, in five cultivars, Qinmei kiwi has the strongest antioxidant effect. In all the experiments, Qinmei kiwi showed even better antioxidative effect than the positive control, vitamin C. This suggested that the antioxidative activity of kiwifruit could not be attributed to the independent contribution of vitamin C, but probably the synergistic effect by two or many components.

By measurement of vitamin C in the brain, it was indicated that treating rats with 12 g kg^−1^ of kiwifruit powder for 21 days was able to induce a significant increase in brain bioavailability. Pb exposure caused oxidative stress resulting in rapid decrease of SOD activity and impairment of mitochondrial function [20]. Interestingly, the primary antioxidant component of kiwifruit, vitamin C brain concentration in treated rats was dramatically reduced following Pb exposure, accompanied by the increase of SOD and GSH-Px activity in hippocampus, thus suggesting a number of bioactive components of kiwifruit were actually consumed for free radical inhibition. These results showed that the antioxidant properties of kiwifruit supplementation are indeed significant to promote neuroprotective and anti-inflammatory effects in Pb poisoning.

Microglia is a new frontier for synaptic plasticity, learning and memory, and neurodegenerative disease research [31]. Microglial cells represent the main source of proinflammatory cytokine production in the central nervous system [32]. Recent evidence indicates aberrant activation of microglia in the hypothalamus and leads to physiological aging, shortened lifespan, and weakening cognition [33]. Indeed it was shown that artificially minimizing glial activation in the hypothalamus increased lifespan and raised the cognitive performance of mice [33]. Pb toxicity on neurons is usually taken into account in studying the Pb-induced cognition impairment [30]. Recently Liu et al. [18] proved that microglia played an important role in Pb-induced learning and memory deficits. Oxidation stress induced by Pb exposure could subsequently activate microglia and thus develop a more inflammatory phenotype. The persistence of an activation of microglia and proinflammatory signal pathway is a potentially detrimental situation favoring learning and memory deficits [18, 34]. In this context, kiwifruit's supplement reduced the oxidative stress and microglia activation and thus improved learning and memory damage by interrupting the pathways. The results suggest a great proposal on preventing and treating of Pb in our diet.

Pb exposure could induce a number of nonlethal effects contributing to the neurological and cognitive effects. Among these effects, dendritic spine loss was considered to be a substrate of cognitive dysfunction, considering the implications of these structures in mechanisms of synaptic plasticity [12, 28, 35]. Recent evidence by Bian et al. [36] showed that dendritic spines were undergoing dynamic changes during development, including rapid spine genesis and significant pruning. In our Pb exposure model, we found a significant reduction in spine density in CA1 compared with the control group. However, kiwifruit administration significantly increased the number of spines in rats following Pb exposure, which was almost resumed to the original level. This result is consistent with other antioxidants which significantly reversed the spine loss in neural diseases [17, 28]. Considering that the treatment began 40 days posterior to Pb exposure, the dendritic spine loss had already happened at this period. It is thus conceivable that kiwifruits supplement rather than prevent acute spine loss and may contribute to the formation of new dendritic spines. In accordance with it, the effect of another antioxidant, vitamin E, on promoting synaptogenesis was also previously proved efficacious in dentate gyrus of treated rats [37]. Thus, kiwifruit did not seem to directly intervene with the detrimental process of Pb but delivered better resilience to organisms to maintain their balance of spine genesis and pruning. Altogether, the high density of dendritic spine indicates an important role of kiwifruits in synaptogenesis promotion and improvement of learning and memory deficits.

**Figure 10:**
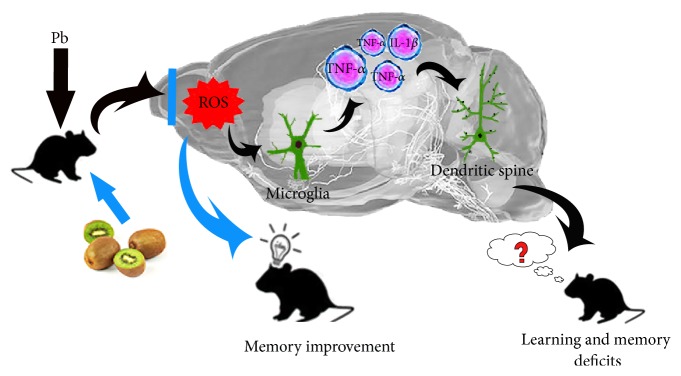
Graphical abstract. In response to oxidative stress induced by Pb, microglia become activated and released cytotoxic factors such as TNF-*α* and IL-1*β*, leading to serious neurotoxic effects including cognition deficit and the dendritic spine loss, while with the supplement of kiwifruit it reduced the oxidative stress and inhibited the microglia activation, manifested as the improved learning and memory ability. Kiwifruit can be a potential complementary diet agent in the prevention and treatment of lead intoxication.

In summary, our findings showed that Qinmei kiwi has the most excellent antioxidative effect among five cultivars. Kiwifruit could improve learning and memory impairment induced by Pb exposure through activation of microglia, thus inhibiting neuroinflammatory and neurodegenerative processes (Figure 10). It is suggested that kiwifruit provides a potential intervention in daily preventive action against chronic Pb intoxication and adjuvant therapy to conventional drugs for Pb intoxication treatment, and more importantly, with a remarkably reduced dosage and adverse effects. But further studies are required to better understand the effects of kiwifruit in the clinical cases, as well as its optimum dosage.

## Figures and Tables

**Table 1:** Primer sequences.

Gene	Primers
SOD2	F 5′-ATTAACGCGCAGATCATGCA-3′
	R 5′-TGTCCCCCACCATTGAACTT-3′
GSH-Px	F 5′-CAGGAGAATGGCAAGAATGA-3′
	R 5′-TCCGCAGGAAGGTAAAGAGC-3′
TNF-*α*	F 5′-AAAGCAAGCAGCCAACCAG-3′
	R 5′-GCCACAAGCAGGAATGAGAAG-3′
IL-1*β*	F 5′-GGGCTGGACTGTTTCTAATGC-3′
	R 5′-TTCTTGTGACCCTGAGCGACCT-3′
*β*-Actin	F 5′- CTGTGCTATGTTGCCCTAGACTTC-3′
	R 5′- CATTGCCGATAGTGATGACCTG-3′

**Table 2:** Vitamin C content in different kiwifruits.

Cultivars	VC content in fresh kiwifruit juice (g/L)	VC content in kiwifruit powder %	Loss %
Wancui	0.29 ± 0.01	0.20 ± 0.08	28.56 ± 0.91
Jinkui	0.30 ± 0.02	0.76 ± 0.14	20.41 ± 1.34
Qinmei	0.28 ± 0.06	0.84 ± 0.18	18.73 ± 0.60
Xuxiang	0.15 ± 0.01	0.48 ± 0.01	20.95 ± 0.97
Hayward	0.18 ± 0.07	0.35 ± 0.06	26.11 ± 0.95

**Table 3:** DPPH radical scavenging activity of different kiwifruits.

Cultivars	DPPH radical scavenging activity (EC_50_)
Vitamin C	5.61 ± 0.25 *μ*g/mL
Wancui	0.75 ± 0.02 mg/mL
Jinkui	0.48 ± 0.03 mg/mL
Qinmei	0.40 ± 0.01 mg/mL
Xuxiang	0.76 ± 0.03 mg/mL
Hayward	0.79 ± 0.01 mg/mL

**Table 4:** FRAP value of different kiwifruits.

Cultivars	FRAP value (mM FeSO_4_ eq./10 mg extract)
Wancui	6.83 ± 0.23
Jinkui	11.10 ± 0.17
Qinmei	11.24 ± 0.24
Xuxiang	5.99 ± 0.16
Hayward	4.56 ± 0.11
